# Skeleton-vasculature chain reaction: a novel insight into the mystery of homeostasis

**DOI:** 10.1038/s41413-021-00138-0

**Published:** 2021-03-22

**Authors:** Ming Chen, Yi Li, Xiang Huang, Ya Gu, Shang Li, Pengbin Yin, Licheng Zhang, Peifu Tang

**Affiliations:** 1 Department of Orthopedics, Chinese PLA General Hospital, Beijing, China; 2 National Clinical Research Center for Orthopedics, Sports Medicine & Rehabilitation, Beijing, China

**Keywords:** Bone, Homeostasis, Osteoporosis, Osteoporosis

## Abstract

Angiogenesis and osteogenesis are coupled. However, the cellular and molecular regulation of these processes remains to be further investigated. Both tissues have recently been recognized as endocrine organs, which has stimulated research interest in the screening and functional identification of novel paracrine factors from both tissues. This review aims to elaborate on the novelty and significance of endocrine regulatory loops between bone and the vasculature. In addition, research progress related to the bone vasculature, vessel-related skeletal diseases, pathological conditions, and angiogenesis-targeted therapeutic strategies are also summarized. With respect to future perspectives, new techniques such as single-cell sequencing, which can be used to show the cellular diversity and plasticity of both tissues, are facilitating progress in this field. Moreover, extracellular vesicle-mediated nuclear acid communication deserves further investigation. In conclusion, a deeper understanding of the cellular and molecular regulation of angiogenesis and osteogenesis coupling may offer an opportunity to identify new therapeutic targets.

## Introduction

Organs in the mammalian skeletal system are inseparable from blood vessels, which function as an ingress-egress meshwork for supplying necessary nutrients and eliminating metabolic wastes.^1^ Within bone, vascular networks are complicated and play critical roles during skeletal development.^2^ In addition to the common consensus that osteoblast-osteoclast equilibrium plays pivotal roles in bone remodeling, essential vascular properties within skeletal structures are also considered modulators of skeletal homeostasis.^3^ Blood vessels and bone are two highly active endocrine organs that regulate neighboring or remote tissues by secreting a multitude of functional molecules.^4,5^ Hence, the osteogenesis (bone formation by osteoblasts) process is not an isolate process but is rather coupled with angiogenesis (the sprouting of new blood vessels from the existing vasculature).^6^

The vasculature within bone is complicated. With a typically stratified organization, the vasculature within bone provides a dynamic niche for skeletal growth and homeostasis.^7^ Owing to their strategic location between the blood flow and bone tissues, endothelial cells (ECs) line the inner layer of vessels to build a bridge between the affluent bloodstream and the bone marrow microenvironment. Researchers have identified various specialized and heterogeneous subtypes of ECs composing bone marrow vascular networks. These vessels exhibit unique endothelial properties, generate distinct metabolic microstates, and perform characteristic functions during bone growth and renovation.^8^ As such, emerging insights are focusing more on delineating the intimate spatial-temporal coordination between angiogenic and osteogenic processes.^9^ During this fascinating interplay, osteolineage cells (osteoblasts, osteoclasts, osteocytes, etc.) release angiogenic factors to regulate vasculature function, while vascular cells (endothelial cells, pericytes, etc.) secrete angiocrine factors to modulate skeletal remodeling in a codependent manner.^10,11^

However, the crosstalk during angiogenesis–osteogenesis coupling process is complex, and the bidirectional roles of endocrine factors in skeletal-vascular homeostasis remain incompletely understood. Therefore, there is an urgent need to explore osteogenesis-angiogenesis coupling. Hence, this review focuses on the following aspects. (1) As bone is a dynamic tissue containing complicated vascular networks, we review the characteristic anatomy of the bone vasculature. (2) The endothelium generally populates the inner cellular lining of blood vessels with an expansive spatial distribution. Here, we summarize the EC heterogeneity of bone vessels in histocytology. (3) Since the vasculature and skeleton function as endocrine organs, they are juxtaposed and interact. We summarize the intimate roles of endocrine factors during the osteogenesis-angiogenesis coupling process. (4) The bone marrow is a complex and dynamic ‘niche’ where hematopoiesis occurs. We review the interaction between bone/vessels and the hematopoiesis system in the bone marrow microenvironment. (5) When favorable molecular communication between the skeleton and the vasculature becomes abnormal, bone development defects and vascular abnormalities may occur. In this case, uncovering certain skeletal and systematic diseases associated with pathological vasculature alterations and probing the detailed mechanisms involved are essential. (6) Since proper vascularization is indispensable for bone formation and remodeling, adaptations targeting the vascular system within bone are desirable. Therefore, we summarize current and novel strategies that synergistically favor vasculature invasion and bone germination for angiogenesis-targeted bone tissue construction. (7) Although tremendous advancements have shed light on osteogenesis-angiogenesis coupling, several questions remain. Consequently, we identify unresolved issues and focus on relevant perspectives, which may provide a fundamental basis for future research and clinical application.

## Anatomy of the bone vasculature

Taking shape from embryonic cartilage rudiments via extensive centrifugal vasculature invasion, bone is a complex tissue with multiple intricate hierarchical architectures.^12^ The presence of a vasculature within bone was described in the 17th century by a prominent scientist, Leeuwenhoek. This phenomenon was further elucidated later in the 20th century by Trueta et al, who found that a blockage in the bloodstream leads to reduced longitudinal bone growth. Early studies have shown that blood vessels within bone are similar in different types of mammals, including rats, rabbits, guinea pigs and humans.^13,14^ The mammalian skeletal system receives ~10%–15% of resting cardiac output, with a few intrinsically avascular exceptions, such as growth plates and articular cartilage.^15^

Normally, bones are categorized into two types: long bones (limb and axial bones) and flat bones (the skull, clavicle and mandible). These bone types are formed via distinct bone formation processes (endochondral ossification and intramembranous ossification).^16^ Flat bones consist of a layer of compact bone. The thickness of flat bones significantly affects the morphology of the blood vessels, and the vasculature also varies in thick and thin parts of the bone. In thinner bones, only periosteal and dural networks exist.^6,9^ In thicker bones, distinct periosteal, cortical, and bone marrow networks can be observed, whose microvascular networks are more similar to those of the long bones. As most studies concern the vasculature in long bones, this review will focus on long bones and the endochondral angiogenesis process. Herein, we divide the current knowledge on vascular microcirculation in bone primarily into two categories: classical theory and novel theory.

### Canonical perspectives on the bone vasculature

In typical long bones, the blood supply generally derives separately from multiple arterial sources, including the central nutrient artery, epiphyseal arteries, and periosteal arteries.^17^ (1) The central nutrient artery consists of a high-pressure system that obtains its blood supply from major systemic arteries. As it penetrates the medullary canal and branches into arterioles, it supplies the whole medullary cavity and the inner 2/3 of the mature bone. Within the dense bone, the blood supply runs through the Harvard and Volkmann canals, entering the medullary cavity with accompanying nerves along the shaft.^18^ (2) Enveloping the bone shaft, the periosteum is a thin bilayer sheath attached to the bone surface. The periosteum is primarily nourished by periosteal arteries beneath the ligamentous attachment; these arteries drain blood from a low-pressure system and supply the outer 1/3 of compact bone and the superficial layer of the cortex.^19^ (3) In the rounded distal end of long bones lies the epiphysis, which covers the articular cartilage. Since the blood supply from the epiphyseal arteries has no direct access to the medullary region, they tend to enter via extensive networks in the peri-articular vascular plexus. This plexus helps maintain isolated blood circulation, which is essential for skeletal growth and forms a ringed band between the joint capsule and growth plate.^20^ Like that in other organs, the vasculature in bone is optimized to exhibit a typical hierarchical structure, from arterial afferent branches to highly branched sinusoidal vasculature networks.^16^ Then, the collection of sinusoidal vessels finally returns to the central nutrient veins, which are located in the central shaft of the diaphysis, along with ascending arteries and nerves.^21^ The other exit channels of the blood flow include epiphyseal, metaphyseal and cortical-periosteal veins, which provide an increased surface area to promote slower flow rates and substance exchange.^22^

### Novel concepts related to the bone vasculature

**Fig. 1:**
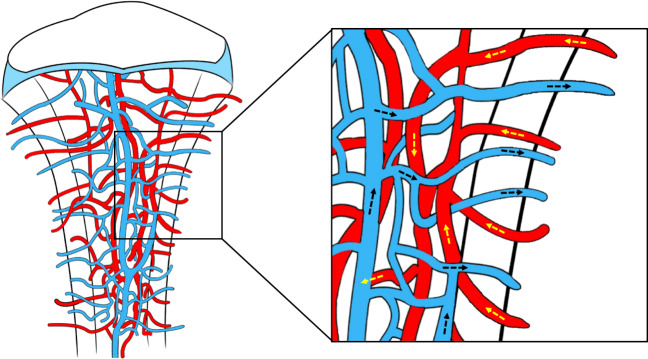
Novel concepts related to the bone vasculature. Transcortical vessels (TCVs) are important vascular structures that originate in the bone marrow and traverse cortical bone canals perpendicularly along the bone shaft, eventually joining the direct periosteal circulation. Over 80% of the arterial blood stream and 59% of the venous blood flow passes through TCVs. TCVs located across the narrow canals of cortical bone provide an orientation for immune cells and hematopoietic stem cells (HSCs) to migrate from the bone marrow to the general outer peripheral circulation and help mediate highly effective blood exchange between the microvasculature in the internal and external circulation

Despite emerging evidence indicating the existence of a conserved blood supply system within bones, the anatomical basis for rapid fluid transport between the general blood circulation and the bone marrow remains unknown. With the development of advanced imaging techniques and big data analysis of the 3D microstructural architecture, previously overlooked vascular structures in murine and human bones have been identified and characterized.^23^ In addition to the known structures, hundreds of blood-filled vessels were discovered to cross the cortical bone perpendicularly along the entire bone shaft. This finding revealed a novel vascular system named transcortical vessels (TCVs) (including arterioles, venules or capillaries), which were postulated to originate from the bone marrow, forming a direct connection between the endosteal and periosteal circulation^24^ (as shown in Fig. 1). In this route, the hard-outer shell of bone creates a barrier for the blood vessel entry-exit route, while osteoclasts in the cutting cone constantly dissolve the calcified matrix and generate transit canals in the cortical bone, thus making blood exchange possible. Supported by the accumulated cross-sectional area of vessels entering or leaving the bone, over 80% of the arterial blood stream and 59% of the venous blood flow passes through TCVs. Hence, blood flow through the long bones is dominated by the contributions of TCVs, while nutrient arteries and large exiting veins play only minor roles.^24^ In this context, a novel theory has been proposed that updates the long-standing viewpoint that the bloodstream trails through the bone marrow with few entry or exit sites.^25^ Significantly, these remarkable structures reveal an integrated closed circulatory system, emphasizing the morphological and functional characteristics of transcortical flow.^21,26^ TCVs located across the narrow canals of cortical bone provide an orientation for immune cells and hematopoietic stem cells (HSCs) to migrate from the bone marrow into the outer peripheral circulation and help mediate highly effective blood exchange between the microvasculature in the internal and external circulation. This may explain the well-known bone hemodynamics phenomenon when peripheral venous access is inaccessible, and the administration of fluids/drugs to the intraosseous space can be performed to restore circulation to the wound under emergency circumstances.^27^ Since the vascular structures within bones are anfractuous, it is expected that new studies will reveal their anatomical characteristics in bone biology and skeletal disease.

## Endothelial cell heterogeneity of bone vessels

Endothelial cells (ECs) populate the inner cell layer of blood vessels in a spatially distributed manner.^28^ ECs participate in multiple physiological processes, including vessel-tissue barrier formation, blood filtration, vasomotor tone maintenance, nutrient trafficking regulation, and immune response control.^29–32^ Generally, EC phenotypes vary across diverse organs, compartments of vascular trees within the same organ, or even neighboring ECs of the same blood vessel.^33^ The characteristics of these heterogeneous subpopulations have been well described in terms of cell morphology, molecular characteristics and gene expression.^34^ For instance, in the vasculature of the heart, liver, lung and kidney, EC properties are organ-specific and gene-distinct. In addition to their shared expression of platelet endothelial cell adhesion molecule-1, PECAM-1/CD31 (a transmembrane glycoprotein that constitutes endothelial intercellular junctions), heart and lung ECs express much more von Willebrand factor (vWF, a glycoprotein that mediates platelet adhesion in the endothelium) but less plasmalemma vesicle-associated protein PLVAP (also called PV1) than kidney and liver ECs.^35,36^

**Fig. 2:**
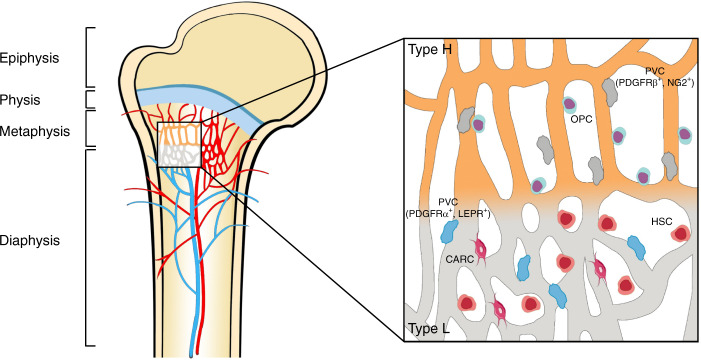
Endothelial cell heterogeneity of bone vessels. Distinct capillary subsets with high heterogeneity in the skeletal system can be subdivided into type H and type L endothelium based on morphology, specialization, molecular identity, and functional properties. Type H vessels are primarily distributed around the endosteum region and metaphysis close to the growth plate. They are linearly arranged with distinctive columnar structures and interconnected with new anastomotic loop-like arches at the distal edge. Type L vessels are located with highly branched networks in the bone marrow region of the diaphysis. Type H capillaries are selectively surrounded by Runx2^+^, collagen 1α^+^, and Osterix-expressing osteoprogenitors, as well as PDGFRβ- and NG2-expressing perivascular cells. Type L capillaries are predominantly infiltrated by PDGFRα- and LEPR-positive cells as well as CAR cells, which interact with HSCs in the regulation of hematopoiesis. Arteries branch into smaller arterioles and flow into type H vessels in the region of the metaphysis near the growth plate. They then converge into a type L sinusoid network at the interface of the diaphysis and are terminally drained via veins located in the contiguous medullary region

In the skeleton, while the frameworks and properties of the interior vasculature are very complicated, much is known about the existence and characteristics of distinct EC subtypes.^37,38^ Capillaries in the mammalian skeletal system have high heterogeneity and can be specifically subdivided into type H and type L subtypes based on morphological specialization, molecular identity and functional properties^8^ (depicted in Fig. 2). Previous studies also revealed the existence of a novel third subset in the murine skeletal system, termed type E vessels, primarily due to its high abundance during late embryonic and early postnatal stages of development.^39^ Here, we mainly discuss these three EC subtypes with heterogeneous functional properties (summarized in Table 1).

**Table 1:** Characteristic data of type H, L and E endothelial cells

Characteristics	Type H	Type L	Type E	References
Marker expression	CD31^hi^EMCN^hi^	CD31^lo^EMCN^lo^	CD31^hi^EMCN^lo^	^39,40^
Location	Endosteum and metaphysis	Diaphysis	Endosteum and compact bone	^40^
Morphology	Linearly structured, columnarly arranged vessels	Reticularly branched network	Linearly structured, columnarly arranged vessels	^21,41^
Surrounding cells	Osterix-expressing osteoprogenitor cells; PDGFR-β and NG2-expressing perivascular cells	Haematopoietic cells; CAR cells; PDGFR-a and LEPR-expressing perivascular cells	Osterix-expressing osteoprogenitor cells	^8,42–44^
Function	Mediate bone formation	Mediate haematopoietic process	Maintain bone formation	^8,39,42^
Blood celocity	High	Low	/	^18,48^
Oxygenation	Well oxygenated	Hypoxic	/	^18,48^
Permeability	Low	High	/	^18,48^
Relationship with aging	Reduction	No significant change	Rapid reduction during postnatal stages	^39^
Interconversion between different ECs	Type H→Type L; Type H→Arterial ECs	Type H→Type L	Type E→Type H; Type E→Arterial ECs	^16,39^

*EC* endothelial cell, *CD31* also known as PECAM-1, platelet endothelial cell adhesion molecule, *EMCN* endomucin, *PDGFR* platelet-derived growth factor receptor, *NG2* neuron-glial antigen 2, *CAR cells* cytokines chemokine ligand 12 (CXCL12)-abundant reticular cells, *LEPR* leptin receptor

### Type H endothelial cells

Type H vessels are characterized by high levels of junctional proteins, including platelet endothelial cell adhesion molecule-1 (PECAM-1/CD31) and sialoglycoprotein endomucin (EMCN) (CD31^hi^EMCN^hi^), and are mainly distributed around the endosteum and metaphysis region.^40^ Type H vessels are linearly arranged with distinctive columnar structures and interconnected by new anastomotic loop-like arches at the distal edge. The leading front of the renascent vasculature exhibits high integrity or low permeability, accompanied by bud-shaped invasions that polarize along the bone elongation axis and orient towards the region of the hypertrophic growth plate.^21,41^ Localized in areas with dynamic bone metabolism regions, type H capillaries are selectively surrounded by Runx2^+^, collagen 1α^+^ and Osterix-expressing osteoprogenitors, as well as platelet-derived growth factor receptor-β (PDGFR-β)- and neuron-glial antigen 2 (NG2)-expressing perivascular cells. These cells, which exhibit a highly positive correlation with the osteoblast lineage niche, synergistically contribute to the osteogenesis process.^8,42^

### Type L endothelial cells

The type L endothelium exists hierarchically downstream of type H vessels and is characterized by discontinuous and fenestrated sinusoidal capillaries. These capillaries display highly branched networks filled with the bone marrow cavity in the region of the diaphysis. With lower expression of CD31 and EMCN (CD31^lo^EMCN^lo^), type L vessels are not associated with Osterix-expressing osteoprogenitors. Instead, they are predominantly infiltrated by cells of hematopoietic lineages.^6^ In addition, two types of perivascular cells localize around type L vessels, namely, cytokine chemokine ligand 12 (CXCL12)-abundant reticular (CAR) cells and leptin receptor (LEPR)-positive stromal cells expressing platelet-derived growth factor receptor-α (PDGFR-α).^43,44^ As such, type L vessels play crucial roles in the regulation of hematopoiesis by secreting combinatorial molecular signals such as stem cell factor (SCF or KITL), CXCL12 and angiopoietin-1 (Ang1).^45,46^

### Type E endothelial cells

Interestingly, type E vessels are a spatially and temporally confined subgroup of capillaries in bone development. These vessels are termed type E because they are highly abundant during late embryonic and early postnatal stages of development, when extensive bone growth occurs. At the molecular level, the expression profiles of type E capillaries were more similar to those of type H than to those of type L endothelial cells and were characterized by higher expression of CD31 but lower expression of EMCN. Due to their high expression of bone morphogenetic proteins (BMPs) and other factors (including Esm1, Kitl, Unc5b, Bcam, Cav1 and Apln), type E vessels are more capable of supporting perivascular Osterix-positive osteoprogenitors and maintaining the balance of regional metabolic properties to sustain osteogenesis.^39^

### Endothelial cell subset variation and interconversion process

The proportions of EC subsets within bone vary greatly during maturation and during the course of aging. Type E vessels represent a small subpopulation of capillaries in newly developing bone, which is abundant during late embryonic and early postnatal stages of development. However, type H and type L capillaries are comparably rare at this stage. Following birth, the proportion of type E ECs decreases with age, while the fractions of type H ECs initially increase during early postnatal life and decline during adulthood and the aging period. Consistent with the high abundance of the sinusoidal endothelium in adult bone, type L ECs increase continuously during postnatal stages and gradually become the major population throughout life.^39^ Moreover, since genetic lineage tracing technology supports the existence of a strict hierarchy of bone ECs, the functional endothelium interconversion process is evolutionarily fine-tuned. As type E ECs occur hierarchically upstream of type H and type L capillaries, type E endothelial cells could give rise to the occurrence of type H capillaries, and type H endothelial cells tend to evolve into type L capillaries. In addition, both the type E and type H subtypes can differentiate into arterial ECs (AECs) during postnatal development, whereas the potential of these subtypes to develop into venous ECs (VECs) remains unknown.^16,39^ Based on the findings presented above, these variable and changing signatures of vascular EC subpopulations are closely correlated in the skeletal maturation and aging process, and highlight a crucial role of the formation of a unique local vascular network during bone development.^47^

### Regional differences in hemodynamics, oxygenation and local metabolic status

Due to the spatial distribution of distinct blood vessel subtypes, blood flow from arteries and distal arterioles traverses a unique landscape to stretch into the capillary sinusoids (since type E vessels are spatially and temporally confined, here, we primarily discuss type H and type L vessels). Normally, the blood flows exclusively into type H vessels of the metaphysis and endosteum. Then, it converges into type L sinusoid networks at the interface of the diaphysis and is terminally drained via central veins located in the contiguous medullary region.^18^ The divergence of local perfusion inside bones results in the creation of regions with unique oxygenation profiles and varying metabolic status. Type H vessels in the metaphysis and endosteum are relatively well oxygenated, while type L vessels situated in the deeper perisinusoidal regions of the diaphysis remain hypoxic due to the lack of a direct arterial supply.^48^ This local difference in vasal permeability and tissue oxygenation status leads to the generation of characteristic metabolic niche microenvironments to meet the demands of osteogenesis. Permeable sinusoids might lead to high reactive oxygen species (ROS) levels, and the eventual hypoxic conditions could support the maintenance of HSCs, thus avoiding damage caused by oxidative stress.^49^

## Coupling of osteogenesis and angiogenesis

The vasculature is generally thought to act as a protective barrier from the harmful external microenvironment and a system for oxygen/nutrient transport.^50^ Interestingly, it has recently been recognized as a very active metabolic and endocrine organ that regulates homeostasis by secreting a multitude of functional substances.^33^ The known roles of the skeleton have also changed from a fundamental organ system providing mechanical support for the body weight to a more crucial reservoir for hormone homeostasis and an endocrine organ for cross-disciplinary interplay with other tissues.^51^ Under this endocrine microenvironment, the establishment of osteogenesis-angiogenesis coupling via molecular regulatory crosstalk between the vasculature and skeleton is important.^52^ As such, vascular cells (ECs, pericytes, etc.) secrete angiocrine factors to mediate systemic skeletal behaviors, while osteolineage cells (osteoblasts, osteoclasts and osteocytes, etc.) release angiogenic factors to help maintain local functions of the vasculature. Downstream of growth signal transmission and gene expression modification, it is important to thoroughly delineate the landscape of the multicellular cascade that mediates osteogenesis-angiogenesis cross-functional interactions.^6,53^

### Osteogenesis promotion by vasculature-derived endocrine factors

Blood vessels are essential for skeletal development via versatile mechanisms. Serving as scaffolds for bone-forming cells and matrix mineralization, blood vessels provide an instructive vascular niche that is necessary for skeletal regenerative activity.^54^ In addition, in response to arrays of extracellular stimuli, ECs or perivascular cells express an altered phenotype referred to as “endothelial cell activation”.^55,56^ They participate in maintaining osteoprogenitor cells that reside in the bone marrow through secretion of substances in an endocrine manner.^47^ These substances are termed angiocrine factors or angiokines; they are released from the heterogeneous vascular lining of bone and act specifically on osteoprogenitors in the metabolically active regenerating callus.^57^ Vascular activation in the bone marrow niche initiates skeletal remodeling processes, thus contributing to the orchestration of bone-vessel interactions (summarized in Table 2).

**Table 2:** Summary of vasculature-derived factors targeting on bone

Factors	Sources	Targets	Effects	Mechanisms	References
BMP	• Endothelial cell	• Mesenchymal stem cell • Chondrocyte	• Promote bone mineralized matrix formation • Promote osteogenic differentiation	• Regulate Smad signaling pathway • Upregulate osteogenic gene expression	^64,66^
DII-Notch-Noggin	• Endothelial cell	• Osteoprogenitor • Chondrocyte	• Promote bone formation • Accelerate the hypertrophy and maturation of chondrocytes	• Restore local expression of protein Sox9	^73,74,76^
RANKL-RANK-OPG	• Endothelial cell; • Osteoblast	• Osteoclast • Endothelial cell	• Regulate osteoclastogenesis process • Maintain endothelial cell integrity and survival	• Regulate NF-κB signaling • Activate PI3K/Akt signal transduction pathway	^79,82^
Sema	• Endothelial cell	• Osteoblast • Osteoclast	• Enhance osteogenic activity • Suppress bone resorption	• Activate Wnt/β-catenin & Rho A signaling pathway	^89,91^
NO	• Endothelial cell	• Osteoblast • Osteoclast	• Modulate osteoblast activity • Keep osteoclast-mediated bone resorption under moderation	• Activate transcription factor NF-κB • Upregulate target genes of NOS2 and IL-1β	^96–98^
PG	• Endothelial cell	• Osteoblast • Mesenchymal stem cell	• Promote differentiation of osteoblast • Mediate BMSC adipogenic differentiation	• Bind with EP4 signal pathway on sensory nerves • Activate Epac-dependent cyclic AMP (cAMP) signaling	^103–105^
Adenosine	• Endothelial cell	• Osteoblast • Osteoclast	• Promote osteoclast differentiation and fusion course • Enhance extracellular matrix formation and osteogenesis process	• Drive oxidative phosphorylation in mitochondria • Activate the PLC-IP3 pathway	^111,113,114^
PTN	• Endothelial cell	• Osteoblast	• Promote osteoblast recruitment and attachment to the extracellular bone matrix • Facilitate fracture healing process	• Integrate with carboxy-terminal domain	^117–121^

*BMP* Bone morphogenetic protein, *Smad* small mothers against decapentaplegic, *Dll4* Delta-like 4, *NF-κB* nuclear factor-kappa B, *PI3K/AKT* phosphoinositide 3-kinase/protein kinase B, *Sema* semaphorin, *NO* nitric oxide, *NOS* nitric oxide synthase, *IL-1β* interleukin 1β, *PG* prostaglandin, *EP4* prostaglandin E receptor 4, *PLC* phospholipase C, *IP3* inositol trisphosphate, *PTN* pleiotrophin

#### Bone morphogenetic protein (BMP)

BMPs are a subgroup of cytokines typically referred to as members of the transforming growth factor­β (TGF­β) superfamily.^58^ In addition to modulating multiple biological processes during embryogenesis, BMPs have also been shown to function as potent osteogenic factors in several clinical applications (including spinal fusion, bone nonunion and alveolar ridge augmentation).^59^ Among the existing subclasses, BMP-2 is ubiquitous in the vasculature, since Bostrom et al. initially observed its existence in atherosclerotic plaques of human arteries.^60^ In another study, Bouletreau et al. found that in bovine capillary and human microvascular ECs stimulated with hypoxia and/or vascular endothelial growth factor (VEGF), BMP-2 mRNA and protein expression was enhanced, with direct effects on bone remodeling.^61^ This provided strong evidence that BMP-2 secreted from ECs could promote bone mineralized matrix formation and bone fracture regeneration by regulating a cascade of cellular processes (including proliferation, differentiation, migration, apoptosis and adhesion) in bone development and repair.^62,63^ BMP-induced osteogenesis is mediated by modulation of the canonical intracellular signaling pathway (homologs of small mothers against decapentaplegic (Smads)) and upregulation of the expression of osteogenesis-related genes (such Runx2, Osx, and Col1a1).^64^ This process helps production of specific proteins (including osteocalcin and osteopontin) and contributes to mesenchymal stem cell (MSC) differentiation, osteoblast commitment and osteogenesis processes.^65^ Studies have shown that Smad7 (an intracellular inhibitor of BMP) is required for both axial and appendicular skeletal development. Smad7 deficiency in mice led to delayed vascular invasion in the growth plate, accompanied by cell cycle impairment in chondrocytes and defects in terminal maturation.^66^ In addition, BMPs are functionally modulated by several extracellular factors (e.g., BMP antagonists). These antagonists include noggin, gremlin, chordin, follistatin, and sclerostin, which act by competing with BMP receptors for binding to ligands.^67^ Among them, BMP-binding EC precursor-derived regulator (BMPER) strongly accelerates bone formation via positive feedback within the BMP2 signaling pathway. However, BMPER alterations resulted in vertebral or rib malformations as well as endothelial thickening and an increase in immature ECs, which implies coupling of angiogenesis and osteogenesis.^68^ Further studies of BMP are needed to reveal the specific cellular and molecular mechanisms involved in the orchestrated interaction of osteogenesis and angiogenesis.

#### Dll4–Notch-Noggin

Previously, endothelial Notch signaling was found to suppress blood vessel growth in various organisms or tissues, such as the retina,^69^ zebrafish embryos^70^ and tumors.^71^ The pathway involved, however, functions as a powerful stimulator of angiogenesis and osteogenesis in the skeletal vasculature.^48^ Delta-like 4 (Dll4) is a critical Notch ligand in bone ECs and a powerful mediator of sprouting and mitosis in the growing vasculature.^72^ Dll4–Notch integration triggers modulated angiocrine secretion of Noggin by ECs, which is an antagonist of growth factors collectively known as BMPs.^73^ Therefore, Noggin regulates the recruitment and differentiation of osteoprogenitor cells, thus promoting bone formation and accelerating the hypertrophy and maturation of chondrocytes in the adjacent growth plate.^74^ Ramasamy et al. once conducted a study on mice with an EC-specific deficiency of Fbxw7 (which mediates polyubiquitination and proteasomal degradation of active Notch); these mice were characterized by overactivation of Notch in ECs. The results showed an increase in type H vessel abundance and bone formation associated with upregulation of flow-modulated gene expression in vascular ECs, including expression of Klf2, Nos3, and Pecam1.^21,48^ However, inactivation of the recombination signal binding protein Jκ (RBP-J) gene affecting Notch-induced transcription not only disrupts vessel arrangement and filamentous pseudopod continuation but also leads to delayed chondrocyte maturation and impaired osteogenic activity. Further studies confirmed that this change in coupling was associated with a decrease in Noggin secretion by ECs.^48,75^ Hence, administering Noggin to Notch-deficient mice rescued the impaired angiogenesis process and mitigated osteogenic defects seen in EC-specific Notch loss-of-function mutants.^48^ The possible molecular mechanism was traced to the ability of Noggin to restore local expression of the protein Sox9. This protein is responsible for initiating cartilage-bone transition and improving VEGF levels, and its blockade leads to impaired skeletal pullulation, hyperinvasive synovial tissue and abnormal ossification.^76^ Since the Notch signaling pathway is well researched during physiological and biological development, the involvement of a cascade of interactions between osteogenic cells and ECs remains unclear and needs further exploration.

#### RANKL-RANK-OPG

The tumor necrosis factor (TNF) superfamily includes osteoprotegerin (OPG), receptor activator of nuclear factor-κB (RANK) and its ligand (RANKL), which have emerged as crucial mediators of bone metabolism.^77^ It is generally recognized that RANKL binds to RANK, and OPG works as an antagonistic endogenous receptor for RANKL. The ligand-receptor interaction transmits specific signals to intracellular components and triggers activation of TNF receptor-associated factors (TRAFs). Then, subsequent nuclear factor-kappa B (NF-κB) signaling leads to the promotion of osteoclast differentiation and the inhibition of osteoblastic bone formation.^78^ RANKL/RANK/OPG factors are also secreted by ECs, and they have a high affinity for osteoblasts and hence coordinate the vascular steady state and bone homeostasis. Upon stimulation with inflammatory cytokines, OPG secreted from ECs could bind with RANKL from osteoblasts to selectively inhibit the osteoclastogenesis process. Similarly, RANKL released by ECs stimulates osteoclast formation during coculture with osteoclast precursors.^79,80^ Romeo et al. showed that ECs at the bone/cartilage interface support a novel nonbone-resorbing osteoclast subtype, termed vessel-associated osteoclasts (VAOs), through the RANKL-RANK signaling mechanism. They found that Tnfsf11a (RANKL) was highly expressed in type H ECs, and endothelial-specific loss of Tnfsf11a (Rankl^iΔEC^) reduced the VAO subset in bones along with a decrease in total osteoclast number. These mutant mice showed an increase in bone mass and an unaltered state of type H ECs, which indicated the importance of RANKL in type H ECs for modulation of bone homeostasis.^41^ In addition, RANK expressed in ECs is involved in the response to paracrine stimulation with RANKL.^81^ RANKL-RANK signaling has been shown to play diversified functional roles in the angiogenesis process, in which it is important in maintaining EC integrity and survival through the phosphoinositide 3-kinase (PI3K)/protein kinase B (Akt) signal transduction pathway.^82^ Furthermore, VEGF upregulates RANK expression on ECs and their precursors during the coupling process, hence enhancing their responsiveness to RANKL, regulating survival effects on ECs, and potentiating RANKL-induced tube formation.^83^ The mechanism of interaction with the RANKL-RANK-OPG pathway has not been fully determined, and manipulation of this process might establish a basis for new therapeutic guidelines for osteogenesis-angiogenesis disorders.

#### Semaphorin (Sema)

Long-term evidence suggests that the semaphorin (Sema) family comprises neuron guidance molecules during embryonic development. These factors provide repulsive or attractive cues for neurocyte migration and growth.^84,85^ To date, Semas have been reported to be involved in several other physiological processes, including bone patterning, angiogenesis evolution, immune responses and cancer progression.^86–88^ Among several subclasses of Semas, Sema3A exhibits the most notable properties in bone metabolism.^89^ Researchers concluded that knockout of the Sema3A gene induced abnormal bone and cartilage development, along with disruptive endochondral ossification and vascular invasion.^90^ Sema3A was proposed to initiate osteoblast differentiation and suppress macrophage colony-stimulating factor (M-CSF)-induced osteoclast activity synchronously in an autocrine or paracrine manner. This process exerts an osteoprotective effect by acting on the Wnt/β-catenin and Rho-A signaling pathways.^91^ In addition, Sema3A has been reported to precede or coincide with blood vessel or nerve fiber invasion into bone, which contributes to skeletal patterning at the spatial-temporal level.^92^ Moreover, Sema3A also regulates EC motility, vessel remodeling and the angiogenesis process by inhibiting integrin function in an autocrine fashion.^93^ In this case, Sema3A and VEGF share common signaling through the transmembrane protein neuropilin 1 (Nrp1), a coreceptor that modulates EC activity and vessel remodeling during angiogenesis. Hence, Sema3A plays an essential role in the VEGF-induced vascular angiogenesis process and in the novel potential crosstalk between the endothelium and bone.^94^ However, the specific mechanisms by which Sema3A is involved in the coupling process remain elusive. Therefore, revealing the relevant receptors and signal transduction pathways will help to facilitate a comprehensive understanding of bone and vasculature homeostasis.

#### Nitric oxide (NO)

NO is a small free-radical gaseous molecule with high diffusivity that conveys essential messages between cells. It activates a range of downstream signals via nitric oxide synthase (NOS) isoforms.^95^ In addition to its classical role in alleviating angina and erectile dysfunction by promoting smooth muscle relaxation and vessel dilatation, NO also plays a crucial role in mediating vascular tone and bone homeostasis by producing endothelial NO synthase (eNOS) in the vascular endothelium.^96^ Since ECs are spatially associated with osteoblasts and modulate the osteogenesis process, research has indicated that NO acts as a mechanoresponsive mediator of EC-osteoblast crosstalk. When cells are exposed to mechanical forces of low strength, the transcription factor NF-κB is activated, and the target genes of NOS2 and IL-1β in ECs are upregulated. Then, increased NOS2-NO production and IL-1β secretion from ECs can enhance the proliferation but decrease the differentiation of osteoblasts. In addition, lipocalin-2 (LCN-2), located downstream of NF-κB activation, could also induce osteoclastogenesis by interacting with RANKL expression and impact interstitial fluid flow within osteocyte canaliculi through fluid shear stress.^97^ However, the effects of NO on the skeletal system are dose dependent and twofold. When eNOS activity is low, NO can stimulate osteoblast activity and maintain osteoclast-mediated bone resorption. However, after iNOS activation at high concentrations, bone loss may occur due to overactivation of osteoclasts and an imbalance in bone remodeling.^98^ An integrated network of ECs and osteoblasts has been demonstrated, but the exact function of ECs in sensing external stimuli is a matter of speculation, and the role of specific adhesion receptors in message sensing remains unclear. When discussing the multifunctional roles of NO in the orchestration of bone and blood vessel development, we need to evaluate the appropriate interactions between these entities. This process may help minimize potential negative effects, resulting in an appropriate balance of vessel-bone turnover course.

#### Prostaglandin (PG)

PGs are lipid metabolites that function in an autocrine or paracrine manner through G-protein coupled receptors (GPCRs) during various physiological processes.^99^ PGs can be produced by ECs in response to injury and mechanical stimuli. Then, they are involved in the regulation of catabolic and anabolic activity during skeletal metabolism.^100^ Prostaglandin E2 (PGE2) is by far the most abundant subtype of prostaglandins released via the activation of prostaglandin E synthases, which functions as multifunctional mediators of bone metabolism involved in both bone formation and resorption processes.^101^ In addition, cyclooxygenase-2 (COX-2) is a notable enzyme secreted from ECs and is responsible for the biosynthesis of PGs.^102^ Pharmaceutical inhibition or deletion of COX-2 impaired osteogenic capability, while exogenous PGE2 rescued conditioned deficiency. This validates the pro-osteogenic effect of PGE2 in regulating bone metabolism and explains why COX2-derived PGs may function as a coupling element for osteoblast and EC crosstalk.^103^ Recently, PGE2 was also shown to facilitate human BMSC adipogenesis instead of pro-osteogenesis (through binding with prostaglandin EP2 and EP4 receptors) and inhibit matrix mineralization (via Epac-dependent cyclic AMP (cAMP) signaling). This helps explain bone loss related to inflammation and metastasis-induced osteolysis.^104^ Another pronounced effect of PGs in vivo is their impact on the vascular system, where PGE2 induces VEGF expression in osteoblastic cells, thus promoting the angiogenesis process. Interestingly, VEGF also promotes PG secretion in turn via activation of specific VEGF receptors to ultimately mediate feedback.^105^ In addition to mediating coupling of bone and vessels, PGE2 also functions as a crucial coordinator of sensory nerves within bones. PGE2 secreted by osteoblastic cells activates sensory nerve receptor EP4 to promote bone formation by inhibiting sympathetic activity through the central nervous system.^106,107^ In addition to its role as the upstream “regulator” in the PGE2-EP4 sensory nerve axis, a newer role as a downstream “effector” was also expounded recently. Bone loss due to sensory denervation or COX-2 knockout was directly attributed to the negative regulation of MSC commitment and was characterized by adipogenesis promotion and osteogenesis inhibition in the bone marrow.^108^ This osteoblast behavior and its molecular mechanism provide the basis of neuro-bone crosstalk for skeletal homeostasis and regenerative therapy. PG functions predominantly in steady-state skeletal growth processes; however, the complexity of PGs makes it difficult to thoroughly clarify the mechanisms involved in endothelial-osteolineage cellular interactions. A better understanding of the effects and roles of PG and other eicosanoids in bone biology is needed.

#### Adenosine

Adenosine is an endogenous purine nucleoside that plays a crucial role in biological energy metabolism and has been implicated in several conditions, such as cardiac circulation, neurodegenerative disorders, immune function, and sleep regulation.^109^ Adenosine carries out its activities via diverse derivatives: adenosine triphosphate (ATP), adenosine diphosphate (ADP) (for energy transfer), and cyclic adenosine monophosphate (cAMP) (for signal transduction).^110^ Recently, emerging insights have focused more on cellular metabolism in physiological processes, which may be mediated by EC-derived intracellularly formed adenosine.^111^ Bone remodeling requires the synthesis of new collagen by osteogenic cells and thus demands a sufficient amount of adenosine, which supports glycolysis as the major metabolic pathway.^112^ Previous studies have shown that osteoclasts differentiate from circulating monocytic precursors to multinucleated giant cells via a specific fusion process. This process is driven by oxidative phosphorylation of ATP and ADP and involves mitochondrial development as indicated by an increase in mitochondria per surface area in osteoclasts.^113^ Another study also showed that ATP was released into the extracellular matrix and enhanced the osteogenesis process by activating the PLC-IP_3_ pathway, accompanied by upregulation of osteoblast genes such as BMP2 and Col3a1.^114^ Moreover, in the angiogenesis-modulating process, adenosine was reported to reduce vascular resistance and increase blood flow to the sternum, as well as enhance EC growth and induce tube formation.^115^ Some factors need to be explored, and the specific adenosine receptors or potential drug effects involved in the osteogenesis and angiogenesis coupling process remain elusive. Further clinical applications of adenosine receptor-targeting treatments in bone-vessel equilibrium are needed to determine the concrete mechanisms.

#### Pleiotrophin (PTN)

Pleiotrophin (PTN), also known as heparin-binding brain mitogen (HBBM) or heparin-binding growth factor 8 (HBGF-8), was initially recognized as a neurite outgrowth-promoting factor present in the rat brain around birth.^116^ PTN is differentially expressed and secreted by bone marrow sinusoidal ECs within the vascular niche. By acting on the N-syndecan receptor expressed on osteoblast precursors, PTN stimulates osteoblast recruitment.^117^ One pivotal osteogenic function attributed to PTN is that it facilitates osteoblast attachment to the extracellular bone matrix by integrating the carboxy-terminal domain, hence promoting the adhesion, migration, expansion, and differentiation of osteoprogenitor cells.^118^ As further verified in several models, PTN was primarily expressed in cell matrices and acted as a target substrate for osteogenesis and matrix deposition. PTN overexpression results in enhanced intramembranous ossification and long-term skeletal growth, which is probably due to increased osteolineage cell recruitment to appropriate regions during bone formation.^119^ In addition, PTN is tightly involved in the fracture healing process. Systemic PTN values show a prolonged increase during the physiological remodeling course of fracture healing, while this elevation is not detected in nonunion patients.^120^ Additionally, a potential regulatory role of PTN in the angiogenesis process has also been reported. Soluble or immobilized PTN has been reported to promote EC proliferation and migration by mediating the stimulatory impact of hydrogen peroxide and endothelial NOS.^121^ PTN also downregulates VEGF-induced stimulatory effects on ECs, partly due to its regulatory role in limiting the excessive angiogenic response.^122^ In all cases, the relevant mechanisms underlying PTN’s involvement in skeletal-vascular homeostasis remain unknown and need further exploration, and the potential applications in clinical situations must be well defined and investigated thoroughly.

### Angiogenesis facilitation via skeleton-derived endocrine factors

Bone has long been recognized as a main structure that supports the body weight, protects internal organs and allows us to move. In addition, it also acts as a reservoir for mineral storage; during this process, osteolineage cells are considered to be efficient “osteoid matrix factories”.^123^ Bone has gradually been revealed to be a dynamic endocrine organ and plays multifunctional roles in homeostasis.^124^ Bone-derived endocrine factors, namely, osteokines, perform highly extraskeletal functions and exert crucial effects on the tissue-specific angiogenesis process.^125^ The metabolic skeletal structure within bone marrow niches could also trigger multisignal responses in a myriad of cell populations (including vascular ECs, chondrocytes, osteoblasts, and osteoclasts).^126^ As a result, bone vessels are reciprocally tuned to ensure a careful homeostatic balance (summarized in Table 3).

**Table 3:** Summary of skeleton-derived factors acting on vasculature

Factors	Sources	Targets	Effects	Mechanisms	References
OCN	• Osteoblast	• Endothelial cell	• Protect vascular endothelial cell functions • Prevent atherosclerosis progression	• Upregulate NO and NF-кB signals • Stimulate PI3K/Akt/eNOS pathway	^127,133,137^
PDGF-BB	• Preosteoclast	• Endothelial progenitor cell • Mesenchymal stem cell	• Enhance capillary abundance • Stabilize tube formation • Mediate osteoblast differentiation	• Trigger PI3K/Akt signaling cascade	^141,144,145^
SLIT3	• Osteoblast	• Endothelial cell	• Promote tube formation and angiogenesis • Help bone remodeling and fracture repair	• Activate ERK mitogen-activated protein kinase and Hippo signaling pathway	^150,151^
VEGF	• Osteoblast • Chondrocyte	• Endothelial cell • Osteoprogenitor	• Promote angiogenesis process • Modulate bone development and homeostasis	• Induced by hypoxia via HIF-dependent pathway • Promote induction of β-catenin and activate Wnt signaling pathway	^156–158,160^
FGF	• Osteoblast • Chondrocyte	• Endothelial cell	• Promote angiogenesis process • Elevate vessel permeability	• Induce EC proliferation, chemotaxis, and intercellular gap-junction communication	^165–167,170^
MMP	• Osteoclast • Osteoblast • Chondrocyte	• Endothelial cell	• Contribute to vascular lumen formation	• Via ERK, AKT, or PI3K and SRC pathways • Release angiogenic factors (VEGF, etc.) from matrix	^176–179,182^
LCN-2	• Osteoblast • Adipocyte	• Endothelial cell	• Make for proper endothelial function • Involved in cardiovascular progression	• Potentiated via IL-1β mediated pathway	^97,184,188,189^

*OCN* osteocalcin, *PDGF-BB* platelet-derived growth factor-BB, *SLIT3* slit homolog 3 protein, *ERK* extracellular signal regulated kinase, *VEGF* vascular endothelial growth factor, *HIF* hypoxia-inducible factor, *FGF* fibroblast growth factor, *MMP* matrix metalloprotease, *LCN-2* lipocalin-2

#### Osteocalcin (OCN)

OCN is the most abundant noncollagenous protein in the bone matrix. As OCN is mainly secreted by osteoblasts during bone formation, it has been shown to function specifically in mineral deposition and skeletal formation.^127^ In addition, it exerts multiple extraskeletal functions that affect other organs and is involved in various physiological processes in an endocrine manner.^128^ These include regulation of insulin and glucose metabolism (beta cells of the pancreas and fat cells),^129^ energy availability and exercise capacity (muscle),^130^ reproductive properties in fertility (testes),^131^ and cognitive function (brain).^132^ More importantly, bone-derived OCN and its effects on the vasculature are another crucial part of crosstalk.^133^ OCN can be primarily divided into two isoforms, undercarboxylated OCN (ucOCN) and carboxylated OCN (cOCN), based on glutamic acid residue carboxylation, which relies on vitamin K.^134^ Among them, ucOCN is predominantly released into the circulation and considered to be associated with bone-vasculature crosstalk.^135^ Multiple lines of evidence indicate an interaction between OCN concentrations and blood vessel function. OCN could exert protective effects on vascular EC functions under specific conditions, hence preventing progression of vascular diseases (such as vascular calcification and atherosclerosis development).^136^ At the molecular level, OCN upregulates NO and NF-кB signals in ECs by stimulating the PI3K/Akt/eNOS pathway.^137^ However, further investigation is needed to determine whether OCN has a direct bioactive role in the vasculature independent of its influence on metabolic outcomes (such as increasing insulin signaling) or whether OCN could serve as a potential marker for vascular diseases.^138^ As such, the specific cellular receptors and underlying downstream signaling pathways in various states need further elucidation, as do the conflicting observations on the OCN-vascular cell interaction, for future therapeutic interventions.

#### Platelet-derived growth factor-BB (PDGF-BB)

The platelet-derived growth factor (PDGF) family is well known for vessel maturation. Among these family members, a subfamily consisting of two polypeptide chains named PDGF-BB has been well documented.^139^ The binding of PDGF-BB (a ligand) to PDGFR-β (a receptor) activates signaling for neovessel formation.^140^ Xie et al. found that PDGF-BB could be released by immature progenitors of osteoclasts (preosteoclasts) on periosteal surfaces. These preosteoclasts play crucial roles in blood vessel growth and osteoprogenitor cell invasion during skeletal development.^141^ Gao et al. showed that macrophage-lineage tartrate-resistant acid phosphatase–positive (TRAP^+^) cells could induce transcriptional expression of periostin and recruit periosteum-derived cells (PDCs) (primarily Nestin^+^ and LepR^+^ cells) to periosteal surfaces by secreting PDGF-BB. The recruited PDCs undergo an osteoblast differentiation process and generate an osteogenic microenvironment coupled with type H vessel formation.^142^ In another study, Su et al. showed that mononuclear preosteoclasts in the subchondral bone of osteoarthritic joints are stimulated after traumatic joint injury. They produce a very high amount of PDGF-BB, which activates PDGFR-β signaling in a paracrine manner to stimulate subchondral bone angiogenesis along with the osteogenesis coupling process.^143^ In addition, treatment with exogenous PDGF-BB or inhibition of cathepsin K (CTSK, a cysteine proteinase highly expressed in osteoclasts) to increase the number of preosteoclasts helps increase type H capillary abundance and attenuate bone loss under osteoporotic conditions.^141,144^ During the process of angiogenesis, PDGF-BB could directly induce endothelial progenitor cell (EPC) activity. Through binding to PDGFR-β, PDGF-BB triggers the phosphoinositide 3-kinase (PI3K)/protein kinase B (AKT) signaling pathway and then promotes re-endothelialization and postnatal neovascularization.^145^ However, deep insights into the molecular mechanisms involved in the osteogenesis-PDGF-BB-angiogenesis chain reaction remain unclear. For example, does increased PDGF-BB production by preosteoclasts occur at the transcriptional or posttranslational level? How is the process initiated during pathophysiologic development? For these purposes, further studies are warranted that involve in-depth investigations of this process and validate the results in different populations.

#### Slit homolog 3 protein (SLIT3)

SLIT3 is a member of a conserved family (SLIT) that was initially discovered in the central nervous system and mediates axonal guidance and neuronal migration through roundabout (ROBO) receptors.^146^ SLITs are widely implicated in multiple pathophysiologic processes, including inflammation development, stem cell differentiation and tumor metastasis.^147–149^ Recently, another role of SLIT3 was explored, and it was found to be involved in angiogenic functions.^150^ In skeletal tissues, research has shown that SLIT3 acts as an osteoblast-derived and Schnurri3 (SHN3)-regulated proangiogenic factor, which increases vascular endothelium levels and evokes osteoanabolic responses.^151^ In the osteoblast-specific inducible SHN3-knockout model, SLIT3 expression was differentially upregulated in bone, and bone mass and type H abundance were drastically increased. In contrast, genetic deletion of SLIT3 led to vascular endothelial decline. SLIT3 deficiency-induced impairment of angiogenesis also provided feedback to the osteoblast niche, followed by impaired osteogenic capacity and reduced bone growth. During this process, bone marrow endothelial progenitor outgrowth cells (EPOCs) administered recombinant SLIT3 showed enhanced vasculature augmentation, as reflected by increased endothelial migration and tube formation capacity via activation of ERK mitogen-activated protein kinase and the Hippo signaling pathway.^151^ In addition, Kim et al. reported that SLIT3 plays a critical role in osteoclast-osteoblast coordinated performance, which links motile bone resorption to bone formation in a temporal-spatial manner.^152^ Mice lacking SLIT3 specifically in osteoclasts displayed low bone loss and significantly reduced type H vessel abundance. This finding further substantiates that SLIT3 secreted from osteoclasts could promote osteoblast capacity and inhibit osteoclast differentiation in an autocrine manner, partially through upregulation of the angiogenesis process. Interestingly, another study conducted by Li et al. addressed the contradictory finding that osteoblasts are the major physiological source of SLIT3 instead of osteoclasts. They found only modest effects of SLIT3 on osteoclast differentiation and no observable bone phenotype alterations when deleting SLIT3 in osteoclasts.^153^ Notably, both osteoblasts and osteoclasts participate in coordinated targeting of the endothelium via orthogonal mechanisms. Therefore, the definite roles of SLIT3 in coordinating bone metabolism and pro-angiogenic functions in concert seem complicated and need further elucidation. Since increasing evidence indicates that the optimal therapy for osteogenesis-angiogenesis disorder requires sequential combination of multiple approaches, SLIT3 may have utility when administered with functional osteoblast- or EC-targeted agents.

#### Vascular endothelial growth factor (VEGF)

The VEGF family includes a range of homologous submolecules that play pleiotropic roles in normal homeostasis and pathological diseases. Among the multiple subcategories, VEGF-A functions as an EC-specific mitogen and master element involved in the angiogenic cascade.^154^ VEGF-A is primarily secreted by hypertrophic chondrocytes and osteoblast-lineage cells, while ECs, osteoblasts and their precursors expressing VEGF receptors (primarily for the tyrosine kinase VEGFR2) vigorously respond to the VEGF signaling pathway.^155^ When VEGF-A integrates with VEGFR2, it induces EC phosphorylation and triggers processes including cell sprouting and proliferation as well as enhancement of vessel permeability, thus promoting development of the vascular system.^156^ VEGF-A commonly exists in three major isoforms, namely, VEGF_120_, VEGF_164_, and VEGF_188_. Among them, the VEGF_164_ isoform is the most crucial variant in proper vascularization and bone-forming activities.^157^ Overexpression of VEGF_164_ in the osteoblast lineage results in elevated bone angiogenesis and osteogenesis through induction of β-catenin. It is then followed by activation of the Wnt signaling pathway, which is terminally accompanied by intensified bone growth and altered morphology.^158^ Conversely, conditional inhibition of VEGF signaling in osteoprogenitors induces an osteoporosis-like phenotype. During this process, vascular invasion of the growth plate is disrupted, leading to increased bone marrow adiposity and repression of endochondral ossification capacity.^159^ Since VEGF is a downstream target of hypoxia-inducible factor (HIF), it has been shown to be induced by hypoxia via a HIF-dependent pathway.^160^ Conditional deletion of HIF-1α in osteoblasts results in impaired VEGF accumulation, strongly reduced type H vessel abundance, and massive osteoprogenitor cell death in the inner hypoxic region of the growth plate. This indicates that HIF and VEGF are indispensable driving forces for inducing angiogenesis during bone formation.^160,161^ Moreover, as osteoblasts not only produce VEGF but also express VEGF receptors, VEGF itself has a direct effect on osteoblasts in the modulation of bone development and homeostasis.^162^ The underlying mechanisms through which VEGF modulates bone vessel pathophysiology are not yet completely understood, posing an intriguing challenge for further research.

#### Fibroblast growth factor (FGF)

FGF is a potent mitogenic group and comprises an extensive family of 18 different ligands integrated with 4 different tyrosine kinase receptors (FGFRs).^163^ In addition to exerting systematic effects on the kidney and parathyroid by inhibiting phosphate resorption and suppressing 1,25(OH)_2_D_3_ production, the FGF family also takes part in maintaining vascular integrity and skeletal function in bone.^164^ FGFs are primarily secreted from chondrocytes and osteogenic cells, while FGFRs, which belong to the tyrosine kinase receptor family, are expressed in the bone vasculature.^165^ Within the group of FGF ligands, FGF-9 has been reported to play a role in the skeletal vascularization process. FGF-9 deficiency leads to impaired neovascularization, reduced hypertrophic chondrocytes, and decreased recruitment of osteoprogenitor cells that participate in bone regeneration. Exogenous FGF-9 not only mediates vascular invasion but also profoundly acts on osteogenesis during the normal repair process.^166,167^ In addition, EC-specific devitalization of genes encoding FGFRs contributes to functions such as vessel permeability elevation, perivascular cell loss, and remarkable abnormalities of the bone vasculature, followed by decreased bone mineral apposition.^49,168^ At the molecular level, FGF directly induces EC proliferation, chemotaxis, and intercellular gap-junction communication, which are involved in skeletal morphogenesis, angiogenesis, and development.^169^ Moreover, the provided data further indicated that FGF also induces the expression of multiple angiogenic molecules (such as VEGFA and VEGFR2) through autocrine and paracrine mechanisms.^170^ In turn, accounting for the mitogenic activity of VEGF, activation of angiogenesis could further contribute to the recruitment of osteoprogenitor cells to participate in bone regeneration. In this context, it would be of great value to investigate the interrelationship between FGF and perivascular cells involved in the embryonic phenotype during skeletal development.

#### Matrix metalloprotease (MMP)

The skeletal system is extensively mineralized and abundant in extracellular matrix (ECM), which is a composite framework of macromolecules (including collagen, enzymes and glycoproteins).^171^ The ECM provides structural and biochemical support to surrounding cells, with substantial mechanical strength and toughness to stabilize the nascent plexuses and maintain bone homeostasis.^172^ MMPs are specific enzymes belonging to the zinc-metalloproteinase family that are implicated in many cellular and pathophysiologic processes, such as cancer metastasis, corneal ulceration, arthritis and vascular disorders.^173,174^ Normally, MMPs induce the proteolytic breakdown of structural components in the ECM space, hence initiating matrix reshaping.^175^ This process of proteolysis is thought to be the first sustained activity in the initial steps of neovascularization and angiogenesis.^176^ MMPs (primarily MMP2, MMP9 and MMP13), mainly secreted by osteoclasts, play crucial roles in skeletal growth and EC specialization. Generally, they promote EC migration and tube formation by proteolytically remodeling the basement membrane due to high gelatinolytic activity, subsequently contributing to vascular lumen formation.^177,178^ The appropriate matrix-cell signaling interaction impacts functional properties and maintains the integrity of the skeletal endothelium via certain signaling pathways (including the ERK, AKT, PI3K and SRC pathways).^179^ Studies have shown that MMP-9 deficiency or endogenous administration of tissue inhibitors of metalloproteases (TIMPs) leads to defective endochondral ossification, diminished ECM remodeling, and delayed vascularization during skeletal healing.^180^ In addition, another study found that MMP-9 plays an important role in osteoclast simulation of angiogenesis, as well as bone remodeling, with both the angiogenic and bone resorptive effects of parathyroid hormone-related protein (PTHrP) being absent in MMP9^−/−^ explants.^181^ In addition, MMPs not only modulate cell-matrix interactions but also regulate the onset and progression of angiogenesis by activating specific angiogenic growth factors and increasing cytokine bioavailability. For instance, since FGF and VEGF are generally trapped in the ECM by various proteoglycans, MMP degradation liberates them to increase their expression levels and allow them to reach their receptors to exert cellular effects related to angiogenesis.^182^ Furthermore, EC-derived MMPs also help resorb the cartilage template, aid in modulating angiogenic blood vessel orientation and direct longitudinal bone growth during endochondral ossification.^41^ Investigations of MMPs help establish that elaborate angiogenesis–osteogenesis coupling occurs through cell-matrix interactions. Since they are important regulators of tissue degradation and cell migration, modulation of these regulators would be beneficial for pathological conditions. Perhaps more significantly, future research should focus more on probing for other essential proteolytic enzymes that are involved in regulating extracellular matrix remodeling and angiogenesis development.

#### Lipocalin-2 (LCN-2)

LCN-2 is a secreted hydrophobic glycoprotein that belongs to a subfamily of small lipophilic molecules in the circulation.^183^ LCN-2 was previously thought to be exclusively secreted by adipose tissue (thought to be a proinflammatory adipokine) and linked to obesity. Recently, it has been reported to be a novel osteokine that is secreted at levels tenfold higher in bone (primarily from osteoblasts) than in white fat tissue.^184^ LCN-2 has been reported to be involved in a range of pathophysiological processes, such as the immune response, apoptosis, infection, inflammation, and energy metabolism. Emerging evidence is available concerning the role of LCN-2 in endothelial function and vascular homeostasis.^185,186^ Augmented LCN-2 expression was found in atherosclerotic plaques and myocardial infarction, which may also mediate the innate immune response in heart failure.^187^ To evaluate the effect of LCN-2 deficiency on endothelium-dependent responses, an LCN2-KO genetically engineered mouse model was developed and showed an amelioration of endothelial dysfunction caused by dietary challenges. This induced higher NO bioavailability, accompanied by enhanced activation of the PKB/eNOS pathway and augmented sensitivity to insulin. On the other hand, administration of exogenous LCN-2 promoted endothelial dysfunction and metabolic insulin resistance by uncoupling eNOS and enhancing COX expression.^188^ In addition, LCN-2 has been demonstrated to be the most upregulated gene in osteoblasts under stimulation with microgravity. The mechano-response is further potentiated by ECs through the IL-1β-mediated signaling pathway to integrate osteogenesis and angiogenesis.^97^ Since LCN-2 could give rise to endothelial dysfunction and cardiovascular disorders, the application of LCN-2 as a dynamic monitor of blood lipid metabolism and a positive marker for the early detection of vascular homeostasis is well targeted. Most studies tend to focus on epidemiological issues related to the improvement of LCN-2 in obesity- and diabetes-related vascular disorders.^189^ Considering that bone is a nonnegligible source of LCN-2, investigating the roles of LCN-2 from osteoblast lineages on vascular cells has become paramount. The crosstalk between the vasculature and bone tissue during the development of endothelial and metabolic dysfunction needs to be further investigated, and the observed biological characteristics also warrant further validation in humans to obtain more convincing clinical data.

## Bone and blood vessels in the hematopoiesis microenvironment

Bone marrow is a complex and dynamic “niche” with multiple functional cell types. The coupling of osteogenesis and angiogenesis is not only crucial for bone formation and vessel sprouting but also important in regulating hematopoiesis.^190^ HSCs populate the niche microenvironment, and their circulation involves leaving the bone marrow, entering the vascular system (mobilization) and returning to the bone marrow (homing).^126^ Improved bone imaging technologies have provided insights into the HSC distribution and confirmed their preferential localization at the osteoblastic surface of trabecular bone as well as adjacent to sinusoidal ECs.^191,192^ The EC-containing vascular zone in the bone marrow functions as an indispensable orchestrator of hematopoiesis, termed the vascular niche.^126,193^ Within the complex niche, ECs mediate HSC self-renewal, mobilization, and homing, with the expression of critical angiocrine factors, including stem cell factor (SCF), C-X-C motif chemokine 12 (CXCL12), and interleukins (ILs).^193,194^ EC-specific deletion of SCF or CXCL12 leads to depletion of HSCs and repaired long-term repopulation activity.^43^ In addition, elevated activation of Notch signaling in ECs not only leads to increased blood flow to the bone but also expands the HSC pool by improving vascular niche function, suggesting that ECs and Notch signaling are critical regulators of HSC activity and cellular polarity.^48,195^ In addition, ECs function as a “backup” niche to support HSC activity and regulate the hematopoiesis process. When the bone marrow is under stress (such as after marrow suppression), the cells outside of the bone marrow medullary space (such as in the spleen) serve to replace bone marrow niche function.^192^

To distinguish it from the vascular niche, the bone microenvironment that harbors the hematopoietic system is termed the osteoblastic niche.^126^ This osteoblastic niche provides medullary canals for the hematopoiesis process, where HSCs are maintained and developing cells of the hematopoietic lineages are retained until they have matured and are released into the vasculature.^126^ Osteoblasts are indispensable for maintaining hematopoiesis within the niche microenvironment. By producing essential factors, such as erythropoietin (EPO), colony-stimulating factors (CSFs), N-cadherin (CDH2), and osteopontin (OPN), they regulate HSC long-term maintenance and quiescence.^190,196^ Among them, EPO is well researched in the hematopoiesis microenvironment. EPO regulates the erythropoiesis process and is a direct target of HIF in osteoblast lineage cells.^197^ Inactivation of prolyl hydroxylase (PHD) or von Hippel-Lindau (VHL) (proteins for degrading HIF) in osteoprogenitors resulted in HIF-dependent activation of EPO, which led to an increase in erythroid progenitors in the bone marrow and spleen and subsequent polycythemia.^198^ Conditional ablation of osteoblasts in mice caused a loss of lymphoid, erythroid and myeloid progenitors in the bone marrow, followed by a decreased number of HSCs and reduced cellularity of bone marrow.^190,199^ In addition, osteoblasts are tightly associated with lymphocyte development and differentiation. Osteoblasts play crucial roles in accelerating B cell progenitor commitment and development via IL-7α. These results indicate that HSCs and other myeloid progenitors might share the same niche and that osteoblasts play supportive roles in regulating multiple hematopoietic lineages.^200^ In general, the coordination between the osteoblastic and vascular niches, with hematopoiesis under different circumstances, is intricate and merits future investigation, which could provide therapeutic approaches for human hematopoietic and bone-related diseases.

## Vessel-Related skeletal diseases and pathological conditions

The skeletal system undergoes uninterrupted remodeling through a lifelong cycle of bone mineral deposition and resorption, which is orchestrated to maintain the precise equilibrium of bone mass accrual. Given the existence of functional and spatial-temporal linkages between osteogenesis and angiogenesis, pathological status is strongly influenced by coupling of these processes.^201^ Since ECs are vital for bone and bone marrow functions, it is necessary to investigate the pathological evidence concerning the vascular system and bones. When favorable molecular communication between the skeleton and the vasculature becomes abnormal, bone development defects and vascular abnormalities start to occur during development.^202^ Several typical pathological diseases are reviewed below (summarized in Table 4), including aging, osteoporosis or osteoarthritis, vascular injury-associated fracture nonunion, EC dysfunction-related necrosis, heterotopic ossification, systemic diabetes mellitus, and osseous neoplasm/metastasis.

**Table 4:** Summary of skeletal and systematic diseases associated with vasculature pathological alterations

Skeletal/Systematic diseases	Potential effects/mechanisms	References
Aging	• Reduction of type H EC quantity and osterix-positive osteoprogenitor abundance. • Accumulation of defective HSCs and alterations in bone marrow stroma.	^8,206,207^
Osteoporosis	• Estrogen decrease causes EC dysfunction, leads to alterations of bone perfusion and bone mass. • Secondary risk factors like glucocorticoid inhibits PDGF-BB, leads to blood flow reduction and osteogenesis impairment.	^141,144,206^
Osteoarthritis	• The preosteoclasts secrete an excessive amount of PDGF-BB, mediating the development of aberrant subchondral bone angiogenesis. • Vascular signal production prompts mTORC1 activation in eroding articular cartilage. • Elevated pro-angiogenic factors mediate inflammatory infiltration, structural damage and nociceptive transmission in OA.	^143,216,218^
Fracture Nonunion	• Abnormal vasculature reduces local bone nutrient sources, increases metabolite deposition and impedes fracture healing efficiency. • Vascular dysfunction exerts detrimental impacts on osteogenic differentiation process and disruption of osteoblast-osteoclast equilibrium. • Inflammatory cells secret and recruit negative regulators towards local injury sites.	^223,225,227^
Avascular Necrosis	• EC damage and grume formation cause blood flow interruption and osteocyte death, articular surface collapse. • Decreased EC abundance and pro-angiogenic factor expression lead to decreased migration ability and increased senescence tendency.	^232–234^
Heterotopic Ossification	• Mesenchymal progenitors function as the major niche in expressing VEGF-A for accelerating ectopic bone formation. • Activation of modulators (BMPs and chondrogenic transcription factors) and loss of mineralization inhibitors (pyrophosphate) lead to HO-vascular calcification.	^236–239^
Diabetes Mellitus	• Microangiopathy causes impaired vasoconstriction and blood flow. • Hyperglycemia diverts BMSCs to a metabolically stressed adipogenic pathway instead of osteogenesis. • AGEs leads to EC dysfunction, pro-angiogenic factor deficiency and a cross-linked imbalance of bone-vessel equilibrium.	^242–244^
Osseous Neoplasm/Metastasis	• Matrix-abundant vascular microenvironment within bone provides fertile soils for metastatic growth. • PDGF-B from vessels provides arteriolar niches for HSCs and DTCs long-term maintaining. • Blood flow decline within bone conduce to interactions between tumor cells and skeletal ECs, thus developing into macrometastasis.	^252–254^

*EC* endothelial cell, *HSC* hematopoietic stem cell, *mTORC1* mechanistic target of rapamycin complex 1, *OA* osteoarthritis, *AGEs* advanced glycation end products, *DTC* disseminated tumor cell

### Aging

People are living longer than ever before, which is a major achievement of modern healthcare. However, this also highlights an unprecedented challenge—aging.^203,204^ Bone adapts its mass and morphology to remodel continuously and exhibits remarkable alterations throughout the lifespan.^205^ During the embryological endochondral bone formation process, osteogenic precursor cells were found to be intimately associated with blood vessel invasion in a pericyte-like fashion. Over time, type H EC fractions were highest perinatally and gradually decreased postnatally during subsequent developmental stages.^206^ During the normal course of aging, type H EC quantity, skeletal blood flow velocity, and associated Osterix-positive osteoprogenitor abundance are drastically reduced, coinciding with a decline in osteogenesis and bone quality. Nevertheless, the total number of ECs in murine bone does not change significantly, owing to an equivalent invariability in sinusoidal type L capillaries.^8^ This helps strengthen the consensus that aging leads to degradation of vessel-forming ECs (mainly type H ECs) and bone-forming osteolineage cells. Thus, type H vessels might serve as a useful marker for aging, which further enforces the existence of a strong stage-specific disequilibrium between osteocyte and endothelial subtypes.^206^ In addition, osteoblastic and vascular niches are complex, and age-dependent microenvironments involve multiple cell populations. Aging is associated with an accumulation of defective HSCs and alterations in bone marrow stroma, which display persistent cell-intrinsic impairment, including DNA damage and reduced functionality.^207^ Manipulation of endothelial properties (such as Notch signaling and HIF pathway reactivity) in aging organisms leads to profound mitigation of skeletal vasculature organization, enhances vascular niche function and helps increase HSC frequency.^195^ This suggests novel insight into targets for the reversal of age-dependent alterations within niches. Accordingly, further study of vascular fusion and bone formation during the aging process may have great clinical value.

### Osteoporosis

Osteoporosis is a systemic disorder that occurs predominantly in postmenopausal women, leading to increased bone fragility and susceptibility to brittle fractures.^208^ During aging, postmenopausal osteoporosis is caused by decreased estrogen levels, resulting in significantly decreased bone mass.^209^ Recent studies have shown that the occurrence of osteoporosis is strongly correlated with significant alterations in the bone vasculature, which is manifested by a marked reduction in intraosseous vascular networks and low bone perfusion.^210^ Vogt et al. found that the blood supply to the lower extremities in the population with osteoporosis or osteopenia is relatively lower than that in healthy people with normal bone mass.^211^ It is worth noting that previous research revealed that the reduction of Type H vessels and associated osteoprogenitors was frequently exacerbated in the ovariectomy mouse model and human patients with osteoporosis compared to age-matched controls.^206^ This provides powerful evidence that skeletal vascular supply and endothelial function are highly correlated with bone mass and osteogenic capacity, and this feature might be an underlying indicator of bone accrual independent of aging. Generally, proper bone formation and angiogenesis processes occur under normal conditions. Favorable vasculature and EC properties ensure that circulating osteoblast precursors and osteoclasts are transported to specific sites, thus contributing to suitable osteogenic processes. When postmenopausal osteoporosis occurs, decreased estrogen may cause EC dysfunction, and this change brings about progressive alterations in the local vasculature, eventually leading to bone loss.^141^

Beyond the solid connection between primary osteoporosis and angiogenesis, secondary osteoporosis, such as glucocorticoid-induced osteoporosis (GIO), must not be ignored.^212^ Indeed, glucocorticoids exert major impacts on vasculature alterations and osteogenesis impairment during bone growth and development.^213^ By inhibiting preosteoclast-secreted PDGF-BB through transrepression of the NF-κB pathway, glucocorticoids lead to a reduction in type H vessel abundance and blood flow supply, accompanied by osteoprogenitor dysfunction. On the other hand, the cathepsin K inhibitor, which targets PDGF-BB secretion from preosteoclasts, has been shown to play a pivotal role in maintaining the osteogenesis-angiogenesis balance in the GIO mouse model.^144^ On the whole, for osteoporosis, future studies should not be limited to the imbalance of osteoblast-osteoclast action. Instead, alterations of blood vessels within bones are a crucial topic, and scholars should take skeletal-angiocrine cytokines and intrinsic interactions into consideration.

### Osteoarthritis (OA)

OA is a chronic degenerative and debilitating noninfectious joint disease that causes degradation of cartilage and abnormal remodeling of subchondral bone.^214^ In addition to pathological features such as inflammatory synovitis, osteophyte formation, and articular cartilage degeneration, aberrant blood vessels breaching the tidemark within subchondral bone have also been reported in OA.^215^ Angiogenesis contributes to the pathogenesis of OA progression, and angiogenic activity perpetuates the development of arthropathies. Aberrant joint subchondral bone angiogenesis was found to develop in preosteoarthritis and early-stage osteoarthritis before joint degeneration occurred.^143^ Preosteoclasts secrete an excessive amount of PDGF-BB, mediating the development of aberrant subchondral bone angiogenesis during osteoarthritis progression. This neovessel formation in the subchondral bone microarchitecture is characterized by the development of osteogenesis-coupling type H vessels, leading to eventual articular cartilage damage and degeneration. Of note, vasculature-secreted signals could improve cartilage chondrocyte mechanistic target of rapamycin complex 1 (mTORC1) activity and trigger VEGF-stimulated subchondral type H vessel formation, eventually leading to subchondral sclerosis and osteophyte formation. Subsequently, this generates positive feedback between OA progression and vascular invasion-mediated interactions.^216^ Another point is that type H ECs (which release MMP and other proteinases) could help digest the cartilage template during longitudinal bone growth, which might facilitate cartilage degeneration in OA.^41^ While angiogenesis was inhibited via suppression of MMP and TGFβ signaling, this approach could reduce type H EC abundance and attenuate articular cartilage degeneration.^215^ In addition, immoderate vascularization also leads to inflammatory infiltration and local pain receptor upregulation and hence mediates structural damage and nociceptive transmission.^217^ Infiltration of inflammatory cells in OA could be driven by elevated levels of proangiogenic factors (including several cytokines, growth factors, and chemokine receptors) to erode subchondral bone and articular cartilage far beyond the OA synovial tissue.^218^ In general, the pathogenic mechanisms of diverse OA subtypes (such as spontaneous aging and metabolic dysregulation-associated OA) could be different and complicated. The specific role of angiogenesis and the complicated coupling mechanisms in OA development should be further studied.

### Fracture nonunion

As one of the most common aspects of traumatic injuries, bone fracture has attracted much attention among different populations.^219^ Under pathological bone fracture conditions, ~10% of patients suffer from delayed healing and disunion, which has long been ascribed to poor mechanical stability, extensive periosteal disruption, steroid hormone abuse, etc.^220,221^ Indeed, bone fracture healing is a multistep and overlapping process that is known to proceed through definable temporal and spatial sequences. This process often involves angiogenesis–osteogenesis juxtaposition and inflammatory infiltration. Thus, emerging insights are focusing more on the disruption of blood vessels and the impairment of mineralized tissue within bones.^222^ Impaired fracture healing is commonly associated with abnormal blood vessel formation, insufficient blood supply, limited nutrient availability, and increased metabolite deposition at the local site of injury, suggesting that an impaired angiogenic response is a major cause of this pathology.^223^ This generally occurs when the fracture is combined with a large number of vascular injuries and eventually increases the risk for delayed healing or nonhealing, owing to a disequilibrium in intercellular signaling crosstalk between osteogenesis and angiogenesis.^224^ In animal experiments, studies have demonstrated that antiangiogenic drug interventions can cause a significant delay in the healing process during the fracture period, and proangiogenic measures (delivery of growth factors such as VEGF, FGF, etc.) are thought to accelerate bone repair and regeneration.^225^ During the fracture repair process, vascular dysfunction has detrimental impacts on the osteogenic differentiation process and disrupts osteoblast-osteoclast equilibrium to accelerate microdamage at the bone fracture site.^226^ In addition, inflammatory cells secrete and recruit negative regulators to local injury sites and impede osteoblast assembly and callus remodeling, therefore making fracture healing a difficult process.^227^ From the perspective of promoting vascularization, the specific mechanisms involved in bone angiogenesis under fracture and healing conditions need further exploration. This would help to pave the way for a novel interaction between several cellular elements and signal pathways that effectively target bone loss and promote fracture repair.

### Avascular necrosis

Sustained perfusion of blood vessels is critical for persistent maintenance and survival of skeletal tissue, the loss of which could lead to vascular dysfunction-related necrosis of bone, such as osteonecrosis.^228^ Avascular necrosis of the femoral head (ANFH) is one of the most common conditions associated with bone vasculature disruption, followed by hip joint destruction.^229^ In addition, osteonecrosis of the jaw (ONJ) is another rare condition where the bone of the lower/upper jaw starves from a lack of blood supply.^230^ Several classical factors tend to cause an increase in susceptibility to avascular necrosis, including microvascular injury, steroid abuse, excessive alcohol consumption, dearticulation, etc.^231^ The disease course of osteonecrosis involves damage to ECs, accompanied by decreased migration activity and an increased senescence tendency, leading to aberrant angiogenic capability for neovascularization and reduced intraosseous microcirculation.^232,233^ This was combined with increased susceptibility to grume formation, which caused an interruption of blood flow in the microvasculature. Then, subsequent ischemia led to the necrosis of osteocytes, the collapse of the bone trabecula and articular surface, a notable impairment of bone strength and subsequent osteonecrosis.^234^ The course of avascular necrosis involves decreased angiogenesis in bone and suggests a potential osteolineage-intrinsic regulatory mechanism for angiogenesis activity under pathological conditions. Considering that avascular necrosis is a result of several biomechanical and biological factors, a more in-depth analysis illuminating the angiogenesis event along with bone or cartilage involvement is fascinating and indispensable, and further evidence is needed to support the angiogenesis-promoting effects.

### Heterotopic ossification (HO)

HO, also known as myositis ossificans, is a benign pathological condition in which the repair process is disturbed and results in extraskeletal bone formation. HO can be a common complication of musculoskeletal trauma or injury to muscle and other soft tissues.^235^ The HO process is highly reliant on angiogenesis that progresses through endochondral ossification to mineralization. Vascular histomorphometry analysis revealed a temporospatial spectrum in which abnormal patterning of vascularity in HO coincides with lesion ossification and maturation. This pattern of vascularization suggests a highly coupled pathophysiologic process involving the coordinated processes of osteogenesis and angiogenesis.^236^ As extremity trauma and inflammation induce a proangiogenic environment that is characterized by an increase in endothelial structure, upregulation of vascular signaling is thought to occur prior to pre-HO chondrogenesis. Interestingly, instead of endothelial lineage cells, mesenchymal progenitors function as a major niche expressing VEGF-A at the burn/tenotomy injury site for ectopic bone formation. In contrast, conditional ablation of VEGF-A in Prx1 lineage cells leads to a marked, clinically relevant reduction in posttraumatic HO formation.^237^ Furthermore, studies have recognized a unique form of HO, vascular calcification, which occurs in the bone microvascular network. Loss of patency and vasomotor function are characterized by dysfunction termed the “microvascular dead space”. This dysfunction is associated with heterotopic bone formation in the vasculature, which is triggered by common inflammatory and metabolic disorders. At the molecular level, it has been widely thought to involve activation of modulators, including BMPs and chondrogenic transcription factors, and loss of mineralization inhibitors, such as pyrophosphate.^238,239^ In summary, substantial advances have been made in elucidating the complicated mechanisms of HO development, but the role of angiogenesis-mediated progression to ossification needs to be further explored. In bone biology, the interconnection between vascular function and heterotopic ossification raises questions about the exact phenotypic impacts of antiangiogenic therapy on common conditions.

### Diabetes mellitus

In addition to protopathic vascular and skeletal pathologies, general systemic diseases also have a detrimental impact on the skeleton and vasculature. Diabetes mellitus (DM) is characterized by hyperglycemia with insulin resistance in peripheral tissues or dysfunctional insulin secretion by pancreatic cells.^240^ Studies have found that DM tends to cause skeletal health disorders, which include a high risk of fracture, delayed bone healing, and diabetes-associated bone mineral loss.^241,242^ Compelling evidence suggests that hyperglycemia diverts BMSCs to a metabolically stressed adipogenic pathway instead of osteogenic responses through monocyte-adhesive hyaluronan matrix synthesis. The increase in adipocytes and decrease in mature osteoblasts consequently lead to demineralization of trabecular bone.^243^ In addition to skeletal comorbidities, microangiopathy and macrovascular-induced morbidity occur simultaneously under diabetic conditions. As significantly altered vascular thickness distributions within healed calvarial defects were observed in Zucker diabetic fatty rats (ZDF, an established rat model for obese T2DM), impairment in capillary bed formation and low perfusion volumes in the medullary cavity have been linked to DM along with osteonecrosis. This helps explain why the interruption of bone formation during T2DM progression is due to a reduction in bone marrow blood flow and impairment of coupling mechanisms linking endothelium-dependent vasodilation to bone cell remodeling.^244^ At the molecular and cellular levels, impaired angiogenesis in diabetes could be partially attributed to senescence-related macroprotein derivatives termed advanced glycation end products (AGEs). These AGEs interact with their receptors (RAGEs), and the AGE-RAGE axis evokes inflammatory activation in various cells. This eventually leads to EC dysfunction, proangiogenic factor deficiency, aberrant progenitor cell mobilization, and an imbalance of the bone-vessel equilibrium.^242,245^ Under DM conditions, hyperglycemia stimulates mitochondrial free radical production and alters the redox balance to promote endothelial dysfunction in the vasculature, which is associated with elevated oxidative stress and uncoupling of endothelial NOS.^246^ In addition, redox-dependent activation of the RhoA/Rho-associated kinase and Src/vascular endothelial cadherin signaling pathways, together with Akt inactivation, also contribute to endothelial barrier dysfunction in diabetic bone marrow niches.^247^ The dynamics and interrelationships between bone regeneration and angiogenesis in the compromised context of DM are challenging to elucidate. Hence, further studies are needed to identify specific factors involved in adverse bone formation and vascularization outcomes in diabetic conditions.

### Osseous neoplasm/metastasis

The skeleton is a common site for tumor development, metastasis and relapse, which often have poor curative effects and low survival rates.^248^ Studies have shown that the vasculature within bones provides a protective niche for cancer cells in the progressive cascade.^249^ This stage initiates with the extravasation and lodging of tumor cells in the matrix-abundant vascular microenvironment within bones. Then, the bone marrow vascular niches provide fertile soil for an interaction between tumor cells and bone ECs, which promotes metastatic ability and supports disseminated tumor cell (DTC) reactivation.^250,251^ Specifically, type H capillaries along with PDGFR-β^+^ perivascular cells express high levels of PDGF-B, providing tissue-specific instructive vascular niches for long-term maintenance of HSCs and DTCs.^252,253^ In addition, blood flow decline within the niches is generally conducive to interactions between tumor cells and skeletal ECs, thus leading to macrometastasis. Based on these findings, the modulation of blood flow along with chemotherapy and radiation prevented cancer cells from acquiring a quiescent phenotype. This was done by reducing pericyte expansion, suggesting a potential paradigm for anticancer treatment in bone metastasis.^253^ Notably, a common type of hematologic tumor in the bone marrow is leukemia, which is a subtype of cancer that starts in the bone marrow niche with abnormal blood cells. In the leukemia microenvironment, stromal cells provide metabolic support to malignant hematopoietic cells, which in turn produce angiogenic factors promoting neovascularization and interactions between malignant cells and their niche.^254,255^ Angiocrine signals help regulate quiescence and therapy resistance in skeletal metastasis; for instance, VEGF activation was found to promote aggressive leukemia cell proliferation, while blocking VEGFR signaling increased the sensitivity and efficacy of chemotherapy.^256^ The existing therapeutic technologies are limited to the detection of bone metastasis in the late stage, which is often less curative. Understanding and targeting the survival, quiescence, migration, and proliferation of tumor cells within bone that precede primary detection during the early stage are essential. Therapeutic regimens that either directly target quiescence-promoting vascular niches or reduce promiscuous interactions between cancer cells and the surrounding microenvironment will hopefully show a powerful ability to delay or even prevent metastatic relapse in bone.^257^

## Angiogenesis-targeted therapeutic strategies

Since proper vascularization is indispensable for bone formation and remodeling, adaptations targeting the vascular system within bone need to be elaborated. The bone vasculature serves an important function by working as an anabolic agent to modulate the function of osteolineage cells and has inspired longstanding strategies for influencing the development of the skeletal system. In recent years, the type H capillary subset has facilitated the investigation of vascularization progression and osteogenesis-promoting effects.^258^ Strategies that target the type H endothelium could lead to the reappearance of osteoprogenitors and the mitigation of bone accrual loss, which might be a good choice for therapeutic approaches in a range of skeletal conditions in human patients.^206^ It is widely thought that manipulation of proangiogenic factors or signaling pathways involved in skeleton-vasculature interactions results in anabolic responses and can be utilized as a meaningful therapeutic target. As such, it is postulated that this approach could help to reverse the pathological process and orchestrate osteogenesis-angiogenesis coupling during the regenerative process.^224^ For instance, localized activation of HIF signaling with small molecule inhibitors that block PHD enzymatic activity (such as deferoxamine mesylate, DFM) could be an appealing strategy to facilitate bone repair. Studies have demonstrated that DFM administration can enhance HIF-1a stability and promote the VEGF-mediated vascularization process. Hence, it contributes to an enhancement of CD31 transcription levels and a substantial expansion of type H EC abundance. This phase eventually increases the vascular callus size and connects the bridging lesions in skeletal fractures.^8,259^

Insufficient vascularization of engineered bone tissue and functional blood vessel deficiency have become the biggest obstacles to clinical application.^260^ With advancements in tissue engineering technology, emerging insights are focusing more on suitable grafts with extensive blood supply or biomaterial scaffolds implanted with crucial angiogenic factors for the repair of bone defects.^261^ In an experiment on the repair of sheep bone defects, arterioles and venules were placed on bone grafts. After 18 weeks, the grafts degraded, and the activity of osteoclasts was observed to be significantly lower, suggesting that angiogenesis is indispensable for bone regeneration.^262^ In most cases, smart biomaterial scaffolds implanted for bone construction tend to serve as multifunctional templates for the establishment of the neovascular system.^263^ When transplanting EPC and MSC coculture models inside scaffolds in vitro, microcapillary-like structures could be formed. This would better facilitate the EC seeding capacity and bone-forming cell germination ability due to the existence of much higher porosity and pore size.^264^ Due to the provision of favorable proangiogenic factors and beneficial microenvironments for osteogenesis, tissue-engineered bone strategies are gaining popularity. These strategies function by promoting early vascularization and restoring blood supply to defect areas, and they have potential for future investigations.

Recently, modern strategies for bone regeneration have focused more on gene therapy. The transfer of genetic material could be performed either in vivo or through in vitro gene-transfer procedures.^265^ Yang et al. reported that aptamer-mediated activation of miR-497~195 posttranscriptionally inhibited the expression of Fbxw7 and P4HTM and maintained Notch activity and HIF-1a protein stability in ECs, resulting in the promotion of type H vessels and the enhancement of bone mass in aged mice.^266^ In addition, Fu et al. found that pharmacological administration of the exogenously expressed ZEB1 gene with a DNA-loaded cationic liposome vehicle could ameliorate angiogenesis-dependent osteoporosis conditions. This facilitates histone acetylation on the Dll4 and Notch1 promoters, thereby directly accelerating their transcriptional activity and upregulating endothelial Notch signaling. ZEB1-packaged cationic liposomes targeting the bone vasculature may have a synergistic or complementary effect when combined with an osteoblast-targeted anabolic substance (such as a parathormone analog or an anti-sclerostin antibody).^267^ Gene therapy methods are showing more efficiency and promise, suggesting a novel strategy for treating age-related bone loss and angiogenesis-dependent osteoporosis.^268^ However, future research should investigate more specific genetic mechanisms relevant to osteogenesis-angiogenesis coupling and provide initial insights into its safe clinical use to promote the coupling process.

## Future perspectives

Remarkable advancements have shed light on the significance of angiogenesis during skeletal remodeling.^269^ Further exploration of the coupling mechanisms between osteogenesis and angiogenesis would help identify novel and alternative strategies to promote vasculature invasion and bone germination synergistically for the treatment of vasculature-related skeletal disorders. However, future research might focus on the following points.

### Further exploration of vessel structure and endothelium heterogeneity

Bone is hierarchically organized, and its structure varies from the macroscale to the nanoscale level, measured from centimeters to nanometers.^270^ Owing to its complexity, imaging of the interior vascular microarchitecture remains difficult. Traditional investigation of the skeletal system was based on bone mass/structure assessment by radiographic methods.^271^ Today, direct improvements with skeletal and vascular multiscale imaging techniques are highly desirable. Future research may focus on advanced imaging technologies that address hierarchical spatial and temporal resolution, which include (1) X-ray-based imaging: high-resolution peripheral quantitative computed tomography (HR-pQCT), synchrotron radiation CT (SR-CT), and X-ray microscopy (XRM); (2) magnetic resonance imaging: ultrahigh-field magnetic resonance imaging (UHF-MRI); and (3) microscopy: light-sheet fluorescence microscopy (LSFM) and confocal and two-photon laser scanning microscopy (CLSM & TPLSM).^23^ As such, next-generation research based on these techniques is anticipated to contribute to an entirely new understanding of bone tissue composition and function. These insights into the early detection of bony compartments for bone-vasculature cells may provide precise interventions for bone disorders in the future.

More importantly, as endothelia within vessels are highly heterogeneous, they maintain unique functions and constitute complicated vascular networks in the local microenvironment. In addition to several endothelial subtypes that have been identified and categorized (the abovementioned type H, type L or type E), some other specific ECs observed on histocytology are also anticipated to be discovered in physiopathologic settings. Therefore, single-cell sequencing technology with a strong emphasis on cellular differences and distinct functions is highly recommended in the future.^272^ By viewing deep genetic sequence information from individual cells in a specific microenvironment, the complex ecology of heterogeneous EC states and cellular functions can be investigated extensively. Through this approach, we can establish novel endothelial subtypes and trace distinct trajectories of specific cell lineages in osteogenesis-angiogenesis coupling.^273,274^

### Novel paradigms of bone-vessel communication

Bone and vessels have been recognized as endocrine organs. However, it remains largely unknown how many factors are derived from the bone or endothelium. In what ways are they secreted, and how do they react to targets? Which portions are able to regulate bone/vasculature itself and other organs/tissues? Therefore, probing and evaluating additional key regulatory factors and novel communication modes involved in angiogenesis–osteogenesis coupling is highly desired. (1) Future research should use cutting-edge technologies (such as multiomics studies) to systematically construct comprehensive databases from transcripts to proteins, including mRNAs, miRNAs, lncRNAs, and proteins as well as metabolites.^275^ The systematic discovery of new biological factors would provide a solid link between bone and the vasculature and a basis for further efficient screening of effective intervention targets under pathological conditions.^276^ (2) In addition to conventional mechanisms, including direct cell-cell contact or secreted molecule transfer, novel communication paradigms should be investigated to reveal the potential mechanism of action. Among them, extracellular vesicles (EVs) are a novel paradigm for substance exchange in cell-to-cell communication.^277,278^ EVs can be internalized and secreted by ECs localized in the inner layer of vascular tubes to act on bone cells and display promising therapeutic potential for the treatment of osteoporosis. Reciprocally, they are also released by osteolineage cells as promoters of EC generation during the angiogenesis process. Thus, the function of EVs as critical bidirectional messengers helps establish refined trafficking of endocrine factors for crosstalk between osteolineage cells and ECs.^279^ Altogether, EV-mediated communication provides a new approach to bone-vessel crosstalk and helps elucidate genetic information. When exploring novel coupling factors and communication modes, EVs should be considered an important element during developmental and regenerative processes.

### In-depth mechanisms of angiogenesis–osteogenesis coupling and pathological alterations

The specific role and mechanism of factors involved in bone-vasculature physical coordination or pathological alterations have been reviewed in this article. However, the functions of other pathways and mechanisms underlying the maintenance of systemic homeostasis are still unclear. Thus, (1) it remains to be fully investigated which bone/endothelium-derived osteokines/angiokines may have regulatory effects on skeletal growth and homeostasis. Further experiments should be performed to confirm those factors using data obtained from “gain-of-function” and “loss-of-function” mouse models via the CRISPR-Cas9/12a system and treatment with anabolic agents, such as parathyroid hormone.^280^ Therefore, during the reciprocal interplay, more focus should be placed on determining the distribution of specific receptors, identifying recipient cell types and elucidating the signaling pathways involved. Additionally, we should clarify the response map of different cell types to bone/vasculature-derived factors in the physiological state and identify the specific regulatory functions of newly discovered and known biological elements. (2) Several clinical and basic studies have found a close correlation between skeletal diseases and vascular system diseases, as we have mentioned above. To date, many existing skeletal and systematic disorders are associated with vascular pathological alterations, and their corresponding mechanisms need further in-depth elucidation. It is essential to dissect more intimately the correlations between skeletal and cardiovascular/peripheral circulatory system diseases and formulate optical signaling networks to reveal the variation patterns in diseased states. The discovery of new mechanisms or targets of bone/vasculature factors in regulating the skeletal/vascular system under pathological conditions could provide a theoretical basis for biomedical research and development (R&D) as well as clinical guidelines.

### Improvement of therapeutic strategies and transformation of clinical practice

Although progressive strategies targeting bone-vasculature tissue construction have been developed, several questions need to be further addressed. (1) The present therapeutic strategies have several limitations. Several angiogenic growth factors (such as VEGF) are degraded and diffuse to other tissues or lead to undesirable side effects during the skeleton-vasculature interaction process.^158^ Therefore, modification of bone-/vessel-related proteins, nucleic acids, or cytokines for drugs is essential to enhance their stable/active properties, minimize undesirable side effects and improve their targeting efficacy as well as therapeutic potential. (2) Recent multimodality research has been limited to animal models; future research should include cohort studies that involve patients with specific skeletal/vascular or systemic diseases. By establishing certain intervention methods to evaluate the potential relevance and specific characteristics in human populations, we could provide a scientific basis for new clinical intervention strategies for skeletal-vascular homeostasis. (3) Gene therapy for angiogenesis-dependent bone regeneration has shown great efficacy and promise. Further efforts should probe for relevant clinical investigations to circumvent possible limitations and achieve safe trials for clinical treatment of ischemic-skeletal disorders in the future.

## Conclusions

Bone is a well-vascularized tissue permeated by hierarchically organized vascular networks. Blood vessels within the mesh networks of bone marrow are composed of distinct endothelial subtypes. These endothelial cells exhibit unique endothelial properties, contributing to distinct metabolic microenvironments and characteristic functions during the bone renovation process. Generally, skeletal modeling and homeostasis depend upon integrative coordination between diverse cell elements populating the bone marrow niche. In addition to the common consensus that osteoblast-osteoclast equilibrium plays a pivotal role in bone remodeling, a suitable vasculature network within skeletal structures is also a missing but essential component. As such, the osteogenesis process should not be viewed as an isolated phenomenon but considered in combination with angiogenesis as a single entity. The intimate spatial-temporal balance of angiogenesis–osteogenesis coupling during both vascular development and bone regeneration has attracted widespread attention. Reciprocal codependency during the interaction process is commonly dependent upon multiple paracrine signals and active pathways in the bone-vessel axis. Osteolineage cells (osteoblasts, osteoclasts and osteocytes, etc.) release angiogenic factors to help regulate local vasculature functions, while vascular cells (ECs, pericytes) secrete angiocrine factors to mediate systemic skeletal behaviors. However, given that a functional linkage exists between the vascular and skeletal systems, pathological disorders show compromised coupling. When favorable molecular communication between the skeleton and the vasculature becomes abnormal, skeletal defects and vascular abnormalities start to emerge. These pathological maturation processes are generally characterized by dysfunctional osteogenic capability concomitant with impaired vascular properties. Moreover, since proper vascularization is indispensable for bone formation or remodeling, adaptations targeting the vascular system within bone could be elaborated. Their role as critical bone anabolic agents to modulate the function of bone cells should be taken into consideration, which will eventually contribute to longstanding strategies related to the development of the skeletal system. Although remarkable advancements have shed light on osteogenesis-angiogenesis coordination, several problems still exist. More in-depth mechanisms and relevant perspectives need to be elucidated and targeted, which may provide a fundamental basis for future research and clinical applications. In summary, the complicated crosstalk of osteogenesis and angiogenesis helps shed new light on tissue-specific vascular networks and skeletal homeostasis. Further investigations searching for cues for therapeutic application in the context of bone matrix and endothelial subtypes might contribute to the development of approaches for clinical cases involving skeletal disorders.
